# Associations between dysbiosis gut microbiota and changes of neurotransmitters and short-chain fatty acids in valproic acid model rats

**DOI:** 10.3389/fphys.2023.1077821

**Published:** 2023-03-22

**Authors:** Jiu-Gen Zhong, Wan-Ting Lan, Yan-Qing Feng, Yin-Hua Li, Ying-Ying Shen, Jia-Heng Gong, Zhi Zou, Xiaohui Hou

**Affiliations:** ^1^ School of Sport and Health, Guangzhou Sport University, Guangzhou, Guangdong, China; ^2^ School of Kinesiology, Shanghai University of Sport, Shanghai, China

**Keywords:** autism spectrum disorder, microbiota, short-chain fatty acids, neurotransmitter, valproic acid

## Abstract

**Introduction:** The microbiota–gut–brain axis plays an important role in the pathophysiology of autism spectrum disorder, but its specific mechanisms remain unclear. This study aimed to explore the associations of changes in neurotransmitters and short-chain fatty acids with alterations in gut microbiota in valproic acid model rats.

**Methods:** The autism model rats were established by prenatal exposure to valproic acid (VPA). The Morris water maze test, open field test, and three-chamber test were conducted to assess the behaviors of rats. 16S rRNA gene sequences extracted from fecal samples were used to assess the gut microbial composition. Gas and liquid chromatography–mass spectroscopy was used to identify short-chain fatty acids in fecal samples and neurotransmitters in the prefrontal cortex (PFC).

**Results:** The results showed that 28 bacterial taxa between valproic acid model rats and control rats were identified, and the most differential bacterial taxa in valproic acid model rats and control rats belonged to metagenomic species and *Lactobacillus intestinalis*. Acetic acid, butyric acid, valeric acid, isobutyric acid, and isovaleric acid were significantly decreased in the valproic acid model rats compared to those in control rats. Five neurotransmitters (threonine, kynurenine, tryptophan, 5-hydroxyindoleacetic acid, denoted as 5-HIAA, and betaine aldehyde chloride, denoted as BAC) were significantly decreased, whereas betaine was increased in the prefrontal cortex of valproic acid model rats compared to control rats. A variety of neurotransmitters (≥4) were correlated with *Pseudomonas, Collisella*, and *Streptococcus* at the genus level, and they were also related to the decrease of short-chain fatty acids.

**Discussion:** According to this study, we can preliminarily infer that gut microbiota or their metabolic productions (such as SCFAs) may influence central neurotransmitter metabolism through related pathways of the gut-brain axis. These results provide microbial and short-chain fatty acid (SCFA) frameworks for understanding the role of the microbiota–gut–brain axis in autism spectrum disorder and shed new light on autism spectrum disorder treatment.

## Introduction

Autism spectrum disorder (ASD) currently affects 1% of the population worldwide (https://www.who.int/news-room/fact-sheets/detail/autism-spectrum-disorders) with a higher prevalence in male individuals (Hodges et al., 2020). ASD is one of the most serious neurodevelopmental disorders, characterized by two core symptoms of impaired social communication, narrow interests, and repetitive behaviors (Hyman et al., 2020). Scientific evidence suggests that ASD is believed to be a result of gene and environmental interactions (Kim and Leventhal, 2015), but to date, the exclusive pathology of ASD remains unclear. In the past few decades, many theories have been developed to explain the pathogenesis of ASD, such as exciting/inhibiting imbalance and immune system dysfunction (Pretzsch and Floris, 2020; Ma et al., 2022). Meanwhile, pathological changes in the monoaminergic systems are also considered to be related to the etiology of ASD (Kuo and Liu, 2022). However, none of these theories have been universally accepted.

Studies have reported that about 9%–91% of individuals with ASD have gastrointestinal symptoms (Leader et al., 2022) and that prominent dysbiosis is observed in the gut microbiome of children with ASD (Pulikkan et al., 2018; Jones et al., 2022). Meanwhile, the symptoms of individuals with ASD can be improved by regulating gut microbiota *via* fecal microbiota transplantation (Kang et al., 2017) or probiotics supplements (Shaaban et al., 2018). Therefore, some researchers argue that gut microbes may play an important role in the occurrence and development of autism (Hughes et al., 2018; Pulikkan et al., 2019; Xu et al., 2019; Bezawada et al., 2020; Ding et al., 2021), and modification of the microbiota may be a potential therapeutic and/or interventional target (Li and Zhou, 2016; Yang et al., 2018; Liu et al., 2022). The gut microbiota might affect brain function through the gut–brain axis (GBA), which provides bidirectional communication mainly involving the immune system, and neuroendocrine, metabolic, and/or vagal nerve pathways (Li and Zhou, 2016; Fowlie et al., 2018; Abdel-Haq et al., 2019). As an endocrine organ, the microbiota not only derives many metabolic products, such as short-chain fatty acids (SCFAs), which are involved in energy balance and metabolism, but also synthesizes and releases several neurotransmitters, the same as found in the human brain (Dinan and Cryan, 2017; Strandwitz, 2018). Elevated blood serotonin (5-HT) levels were the first biomarker identified in autism research (Gabriele et al., 2014). A cohort study by Dan et al. (2020) found that children with ASD (aged 2–13 years) and chronic constipation (C-ASD) displayed decreased diversity, depletion of species of *Sutterella*, *Prevotella*, and *Bacteroides*, and dysregulation of associated metabolism activities (Dan et al., 2020). The metagenomic analysis also revealed that the differential metabolites between C-ASD and healthy groups were involved in the metabolic network of neurotransmitters, including serotonin, dopamine, histidine, and GABA (Dan et al., 2020). However, there are still no studies on the relationship between gut microbes, short-chain fatty acids, and neurotransmitters in the central nervous system.

A previous study revealed that valproic acid (VPA) has adverse effects on pregnant women and alters the developmental patterns of the embryo by altering 5-HT, GABA, etc. (Sharma et al., 2022). A typical rodent model of autism with environmental/epigenetic origins may better represent the many cases of idiopathic autism than transgenic models carrying mutations in single autism-associated genes (Tartaglione et al., 2019). Furthermore, the gut microbes of the VPA model were similar to those of the autistic population (Liu et al., 2018), and the VPA model was similar to the autistic population in terms of anatomy, pathology, etiology, and behavior (Schneider and Przewlocki, 2005). Therefore, the VPA model is a reliable rat model of autism (Qi et al., 2021) and a valuable tool to investigate the neurobiology underlying autistic behavior (Nicolini and Fahnestock, 2018; Tartaglione et al., 2019). Prior studies showed that the prefrontal cortex (PFC) is a potential locus for autism pathology in different etiology models, including the VPA model (Brumback et al., 2018). However, direct evidence of the neurotransmitters in the prefrontal cortex being altered and gut microbes and/or their metabolites SCFAs in this model is still lacking.

On the basis of the aforementioned studies, we hypothesize that the altered microbiota and its derived metabolic SCFAs may disturb the neurotransmitter system in the PFC by using ASD model rats with exposure to VPA in the prenatal period. This study aimed to investigate the associations of changes in microbiota and SCFAs with changes in neurotransmitters in the prefrontal cortex of autistic rats.

## Materials and methods

### Animals

Pregnant Sprague–Dawley (SD) rats were purchased from the laboratory animal center of Southern Medical University, China. The experimental protocol was approved by the Ethics Research Committee of Guangzhou Sport University and conducted in accordance with the Guide for the Care and Use of Laboratory Animals (NIH Publication No. 85–23). The female rats were either administered 400 mg/kg of valproic acid sodium salt (VPA, P4543, Sigma-Aldrich, United States) (the offspring to be the ASD model rats, denoted as the VPA group) or normal saline (the offspring to be the control rats, denoted as the CON group) by intraperitoneal injection 12.5 days after conception, as previously described (Schneider and Przewlocki, 2005). The rats were housed individually, given free access to food and water, and were allowed to raise their own litters. Litters were not culled, except for the litters prepared for growth and behavioral development tests. The offspring were weaned on postnatal day (PND) 23; then, four rats were housed in a cage. The number of rats was set to 16 in each group and 4 in each cage, according to sex (male: female = 8: 8). The light cycle was 12 h/12 h day and night, and the room temperature was maintained at 23°C ± 2°C. After the behavior tests, all rats were decapitated under anesthesia (administered 40 mg/kg pentobarbital by intraperitoneal injection), and the prefrontal cortex samples were harvested.

### Behavior testing

Morris water maze (MWM) test: The MWM test was performed to assess spatial learning and memory ability using a cylindrical water pool (a diameter of 120 cm and height of 50 cm) filled with water (a water depth of 30 cm and water temperature about 24°C) with a video capture system (XinRuan Co., Ltd., Shanghai, China). The pool was hypothetically divided into four quadrants. All the test rats were trained for four consecutive days before the experiment, and training was performed four times per day at the same times each day. During training, the experimenter placed a platform hidden 1 cm below the water surface in one of the quadrants. Next, for each rat, the experimenter placed the rat on the platform and then moved the rat into water in any other quadrant. The rat was given 120 s to search for the platform. If the rat found the platform successfully, the experimenter let it stay on the platform for 30 s. If not, the experimenter guided it to the platform and let it stay on the platform for 30 s. On the fifth day, which was the test day, the experimenter removed the platform from the pool and placed each rat into the water from one of the quadrants other than the quadrant where the platform was located during training (all rats in the experiment entered the water from the same quadrant). Within a period of 120 s, the experimenter recorded the distance in the target quadrant, duration in the target quadrant, number of the target quadrant crossing, and latency to reach the target quadrant.

Open field test: Each rat was individually put at the center of a box (50 cm × 50 cm × 50 cm). The rat was allowed to explore the box for 5 min, and the distance traveled, distance traveled in the center, static time in the corner, and the number of visits to the center were recorded. A solution of 75% alcohol and water was used to clean the box after each test.

Three-chamber social test: The test was performed in a white three-chamber box (50 cm × 50 cm × 50 cm). All the test rats were placed in the test room for a day to adapt to the surrounding environment before the experiment began. The chamber box was wiped with alcohol to disinfect and eliminate odors before each trial. First, each test rat was placed in the middle chamber of the three-chamber box for 5 min, with one empty cup located in each of the two side chambers. The rats could access both side chambers freely to explore. Then, a stranger rat (S1) was placed in the cup of the left chamber, and a 10-min video recording was taken to track the rat’s activities and trace its trajectory. Finally, another stranger rat (S2) was placed in the cup of the right chamber, and the video recording was continued for another 10 min to track the rat’s activities and trace its trajectory. The durations the rat spent in the S1 chamber, object chamber (Ob, the chamber with the empty cup), and the S2 chamber were recorded.

### 16S rRNA gene sequencing

Fecal samples, 100 mg each, were collected from the rats in PND 23 and then immediately frozen and stored at −80°C before analysis. The fecal samples were used to extract total genome DNA using the DNA extraction kit (MagPure Soil DNA LQ Kit, China) and NanoDrop instrument (ND-2000, Thermo), and the DNA extraction quality was checked by 1.2% agarose gel electrophoresis. The 16S rRNA gene V3–V4 region-specific primers were 338F: ACT​CCT​ACG​GGA​GGC​AGC​A and 806R: GGACTACHVGGGTWTCTAAT. The polymerase chain reaction products of sterile water were considered the negative control for the 16S rRNA sequence and were purified using Vazyme VAHTS™ DNA Clean Beads (N411-01, China). Then, the Quant-iT PicoGreen dsDNA Assay Kit was used to quantify the polymerase chain reaction products obtained using a microplate reader (BioTek, FLx800). The sequencing library was prepared using the TruSeq Nano DNA LT Library Prep Kit from Illumina and was subjected to final fragment selection and purification by 2% agarose gel electrophoresis. Before sequencing on the computer, the library was quality-checked on the Agilent Bioanalyzer using the Agilent High-Sensitivity DNA Kit, and then, the Quant-iT PicoGreen dsDNA Assay Kit was used to quantify the library on the Promega QuantiFluor fluorescence quantification system.

Raw data were analyzed by Metware Biotechnology Co., Ltd. (Wuhan, China). The data analysis, principal coordinate analysis (PCoA), and alpha and beta diversities were calculated by the breakaway method implemented in Quantitative Insights Into Microbial Ecology (QIIME) version 2.0.

### SCFA identification

Sample preparation: An amount of 20 mg of the fecal sample (collected in PND 23) was accurately weighed and placed in a 2-mL EP tube. Then, 1 mL phosphoric acid (0.5% v/v) solution and a small steel ball were added to the EP tube, and the mixture was ground for 10 s at 20 Hz three times, vortexed for 10 min, and ultrasonicated for 5 min under an ice bath (the vortex meter frequency was adjusted to the maximum for all vortexing operations). Next, 100 μL of the supernatant was added to a 1.5-mL centrifuge tube after the mixture was centrifuged at 12,000 r/min for 10 min at 4°C, and 500 μL of the MTBE (containing internal standard) solution was added, vortexed for 3 min, ultrasonicated for 5 min under an ice bath, and centrifuged at 12,000 r/min for 10 min at 4°C. The supernatant was collected and stored in a refrigerator at −20°C until GC-MS/MS analysis. An Agilent 7890B Gas Chromatograph coupled to a 7000D mass spectrometer with a DB-5MS column (30 m length × 0.25 mm i.d. × 0.25 μm film thickness, J&W Scientific, United States) was employed for GC–MS/MS analysis of sugars. Helium was used as the carrier gas at a flow rate of 1.2 mL/min. Injections were made in the splitless mode, and the injection volume was 2 μL. The over-temperature was maintained at 90°C for 1 min, raised to 100°C at a rate of 25°C/min, raised to 150°C at a rate of 20°C/min, maintained for 0.6 min, raised to 200°C at a rate of 25°C/min, maintained for 0.5 min, and finally, allowed to run for 3 min. All samples were analyzed in multiple reaction monitoring modes. The injector inlet and transfer line temperatures were 200°C and 230°C, respectively.

### Neurotransmitter identification

The prefrontal cortex samples (the anterior 1 mm of the forehead was cut with a surgical blade, collected in PND 28) were removed from the −80-C refrigerator and thawed on ice. After thawing, the samples were weighed and placed in a 2-mL EP tube. A small steel ball was added and homogenized four times at 30 Hz, each time for 30 s (if the sample was not easy to weigh, part of it was placed in the EP tube first and then homogenized to weigh 50 mg). After homogenizing, 500 μL of 70% methanol (methanol: water = 7:3, V: V) solution was added. The centrifuge was run at 12,000 r/min at 4°C for 10 min, and then, 100 μL of the supernatant was removed for LC-MS/MS analysis. The analytical conditions were as follows: high-performance liquid chromatography (HPLC): column, Waters ACQUITY UPLC HSS T3 C18 (100 mm × 2.1 mm i.d., 1.8 µm); solvent system: water with 0.1% formic acid (A) and acetonitrile with 0.1% formic acid (B); gradient: started at 5% B (0 min), increased to 95% B (0–8 min), maintained at 95% B (8–9.5 min), and finally ramped back to 5% B (9.6–12 min); flow rate: 0.35 mL/min; temperature: 40°C; and injection volume: 2 μL.

### Statistical analysis

The independent sample *t*-test was used to compare the variables of the two groups. Pearson correlation analysis or Spearman correlation analysis was conducted when appropriate. Meanwhile, summaries of the taxonomic distributions of operational taxonomic units (OTUs) were constructed to calculate the relative abundances of gut microbiota at different levels. Three different parameters (observed_OTUs, and Shannon and Simpson indices) were used to assess the alpha diversity. Distance matrices (beta diversity) between samples were assessed using principal coordinate analysis (PCoA). The random forest algorithm was utilized to find the key discriminatory OTUs. The linear discriminant analysis effect size (LEfSe) was further used to identify the dominant bacterial taxa in both CON rats and VPA rats. A *p*-value of less than 0.05 was considered statistically significant.

## Results

### VPA rats express autism-like behaviors

The behavior results are shown in Supplementary Material S1. From the MWM test, the duration in the target quadrant and the number of the target quadrant crossing of the VPA group were significantly decreased compared to the CON group (VPA: 25.6 ± 6.1 s versus CON: 36.7 ± 5.9 s, *p* < 0.001, *t*-test, Supplementary Figure S1B; VPA: 9 ± 2 versus CON: 16 ± 3, *p* < 0.001, *t*-test, Supplementary Figure S1C), but the distance in the target quadrant and the latency to reach to the target quadrant were not significantly different between the two groups (VPA: 808.2 ± 226.2 cm versus CON: 849.8 ± 137.4 cm, *p* = 0.534, *t*-test, Supplementary Figure S1A; VPA: 7.0 ± 3.4 s versus CON: 5.0 ± 1.9, *p* = 0.054, *t*-test; Supplementary Figure S1D).

The results of the open field test showed that the total travel distance and the number of visits to the center were significantly reduced in the VPA group (VPA: 4563.9 ± 1383.9 cm versus CON: 17,056.0 ± 6539.6 cm, *p* < 0.001, *t*-test, Supplementary Figure S2A; VPA: 0 ± 0 versus CON: 2 ± 2, *p* = 0.002, *t*-test, Supplementary Figure S2D), the static time in the corner was significantly increased in the VPA group (VPA: 212.2 ± 24.9 s versus CON: 120.8 ± 37.4 s, *p* < 0.001, *t*-test, Supplementary Figure S2C), and the distance traveled in the center showed no significant difference between the two groups (VPA: 541.0 ± 340.2 cm versus CON: 709.4 ± 574.5 cm, *p* = 0.321, *t*-test, Supplementary Figure S2B).

Compared to the CON group, the results of social ability in the three-chamber social test showed that the VPA group’s residence time in the chamber of stranger rat 1 (S1) and the chamber of the object (Ob), and the total time of social exploration were significantly reduced (VPA: 144.2 ± 41.2 s versus CON: 232.9 ± 61.83 s, *p* < 0.001, *t*-test; VPA: 53.2 ± 35.0 versus CON: 141.8 ± 45.6, *p* < 0.001, *t*-test; VPA: 197.5 ± 55.5 s versus CON: 374.7 ± 67.8 s, *p* < 0.001, *t*-test, Supplementary Figure S3A). Regarding the social preferences identified by the three-chamber social test, the VPA group’s residence in the chamber of stranger rat 2 (S2) and the total time of the social interaction (S1 + S2) were significantly decreased, whereas the residence in the chamber of the S1 rat was not significantly different compared to that of the CON group (VPA: 112.8 ± 48.7 s versus CON: 192.1 ± 58.8 s, *p* < 0.001, *t-*test; VPA: 279.5 ± 56.9 versus CON: 344.0 ± 94.4, *p* < 0.001, *t*-test; VPA: 166.6 ± 42.7 s versus CON: 152.0 ± 77.9 s, *p* = 0.224, *t-*test, Supplementary Figure S3B).

### Comparison of gut microbiota, SCFAs, and neurotransmitters between the VPA and control rats

**FIGURE 1:**
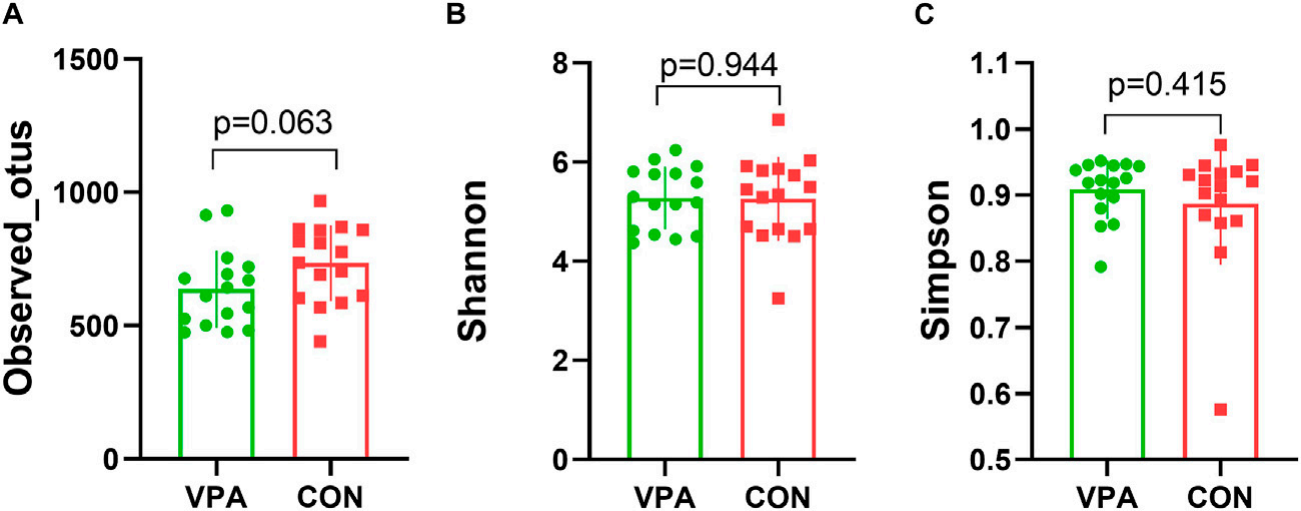
Alpha diversity of the VPA and CON group rats. **(A)** Observed_OTUs; **(B)** Shannon index; **(C)** Simpson index. *n* = 16/16, *t*-test.

**FIGURE 2:**
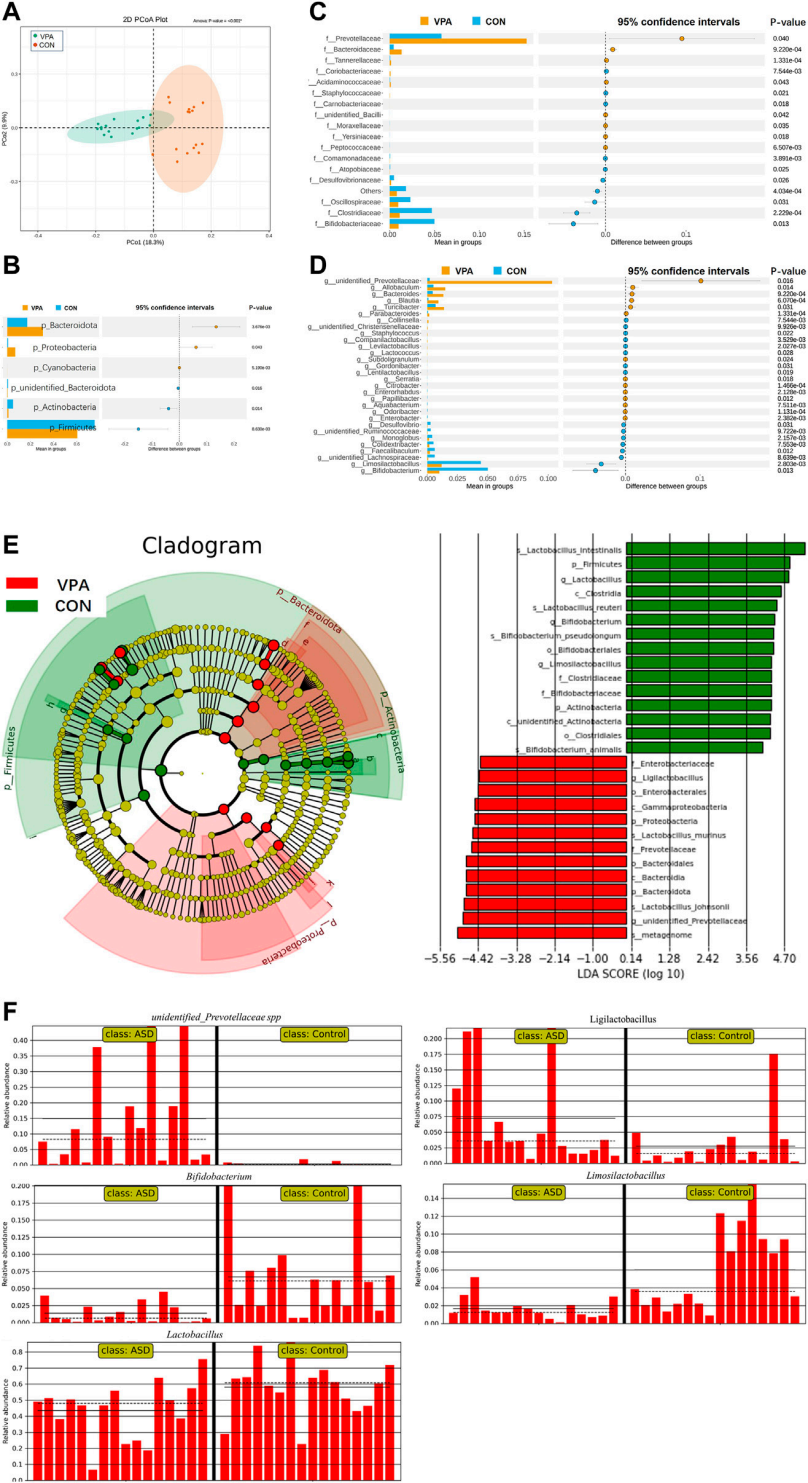
Significant alteration in gut microbiota in VPA model rats compared to control rats. **(A)** PCoA showed a difference in gut microbiota composition between the ASD and CON groups; **(B)** six differential bacterial taxa at the *phyla* level; **(C)** total of 18 differential bacterial taxa at the family level; **(D)** total of 30 differential bacterial taxa at the genus level; **(E)** cladogram and LDA score of LEfSe analysis; **(F)** relative abundance diagram. *n* = 16/16.

The results of gut microbiota composition comparison showed that there was no significant difference in alpha diversity between the two groups (observed_OTUs, *p* = 0.063, Figure 1A; Shannon index, *p* = 0.944, Figure 1B; Simpson index, *p* = 0.415, *t*-test, Figure 1C). However, the principal coordinate analysis based on weighted UniFrac distances shows a significant difference between the two groups (Figure 2A). Further analysis found that six phyla, namely, Bacteroidota, Proteobacteria, Cyanobacteria, unidentified_Bacteria, Actinobacteria, and Firmicutes (Figure 2B), 18 differential bacterial taxa at the family level (Figure 2C), and 30 bacterial taxa at the genus level (Figure 2D) were significantly changed. LEfSe analysis (Figure 2E) found that there were 28 bacterial taxa with statistically significant and biologically consistent differences (13 in the VPA group and 15 in the CON group). These bacterial taxa were the key phylotypes responsible for the different gut microbiota between the VPA and CON groups. As shown in the relative abundance diagram between groups (Figure 2F), compared with the CON group, the abundance of *Bifidobacterium*, *Lactobacillus*, and *Limosilactobacillus* at the genus level in the ASD group was significantly reduced, while the abundance of *unidentified_Prevotellaceae* spp., and *Ligilactobacillus* was decreased.

We further assessed the function of gut microbiota based on the abundance of labeled gene sequences in samples by Genetic Investigation of Communities with Reconstruction of Unobserved States (PICRUSt2) software. The results of the KEGG pathway in level 2 showed that endocrine and metabolic disease (*p* = 0.002), infectious disease: parasitic(*p* = 0.003), infectious disease: viral (*p* = 0.007), poorly characterized (*p* = 0.007), endocrine system (*p* = 0.008), metabolism of other amino acids (*p* = 0.008), lipid metabolism (*p* = 0.009), cell growth and death (*p* = 0.020), and infectious disease: bacterial (*p* = 0.028) were significantly affected in the VPA group (the results are shown in Supplementary Material S2).

### Differentially expressed SCFA levels in the VPA group

**FIGURE 3:**
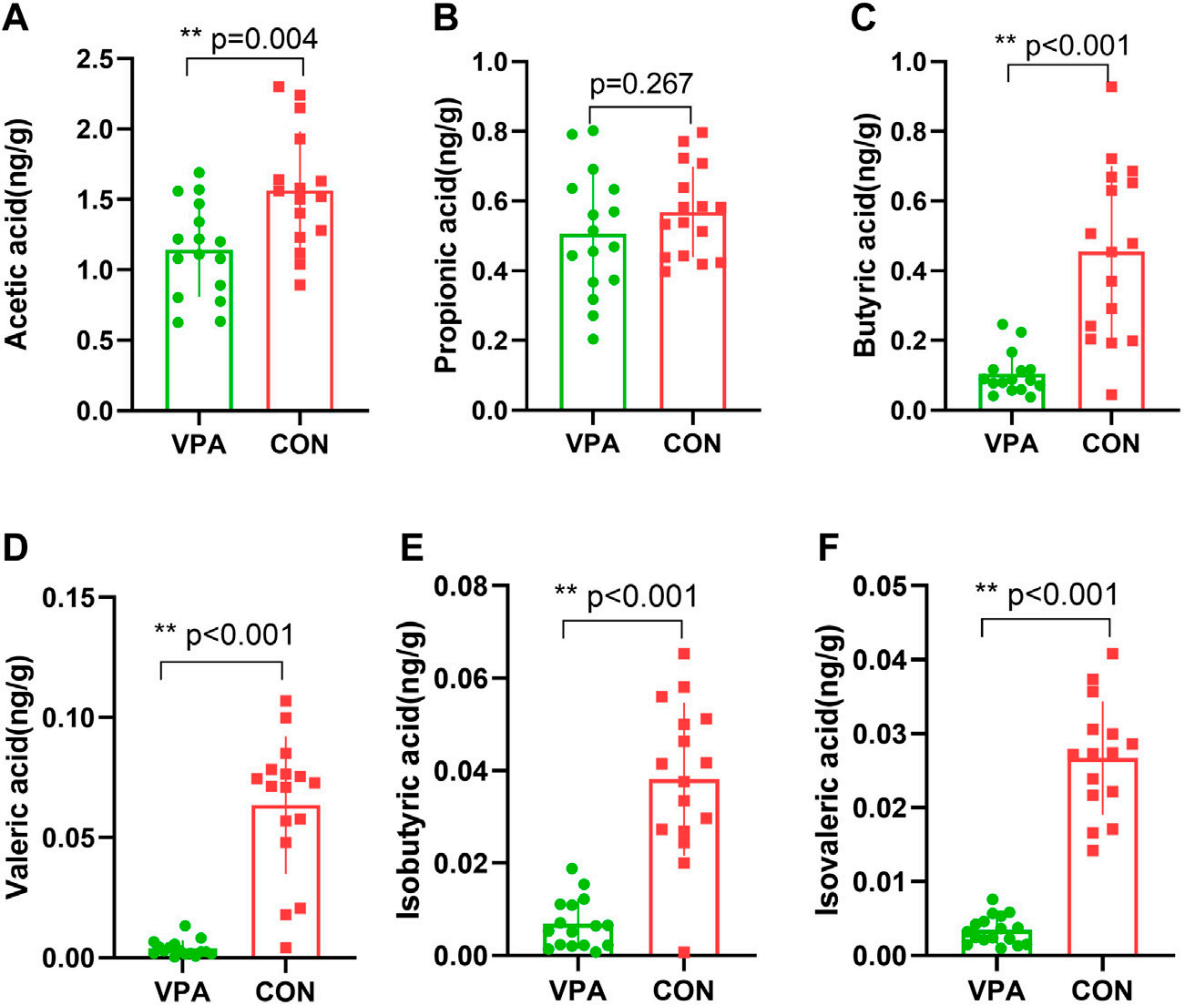
Differential SCFAs in VPA model rats. **(A)** Acetic acid; **(B)** propionic acid; **(C)** butyric acid; **(D)** valeric acid; **(E)** isobutyric acid; **(F)** isovaleric acid. *n* = 16/16.

SCFAs are a major class of key bacterial metabolites that are important for human health. In this study, seven major SCFAs (acetic acid, propionic acid, butyric acid, valeric acid, hexanoic acid, isobutyric acid, and isovaleric acid) were measured in fecal samples. All SCFAs were successfully identified, except hexanoic acid, in the VPA group. The results showed that the levels of acetic acid (*p* = 0.004, Figure 3A), butyric acid (*p* < 0.001, Figure 3C), valeric acid (*p* < 0.001, Figure 3D), isobutyric acid (*p* < 0.001, Figure 3E), and isovaleric acid (*p* < 0.001, Figure 3F) were significantly decreased in the VPA group, whereas there was no significant difference in the level of propionic acid between the two groups (*p* = 0.267, Figure 3B).

### Differentially expressed neurotransmitter levels in the VPA group

**FIGURE 4:**
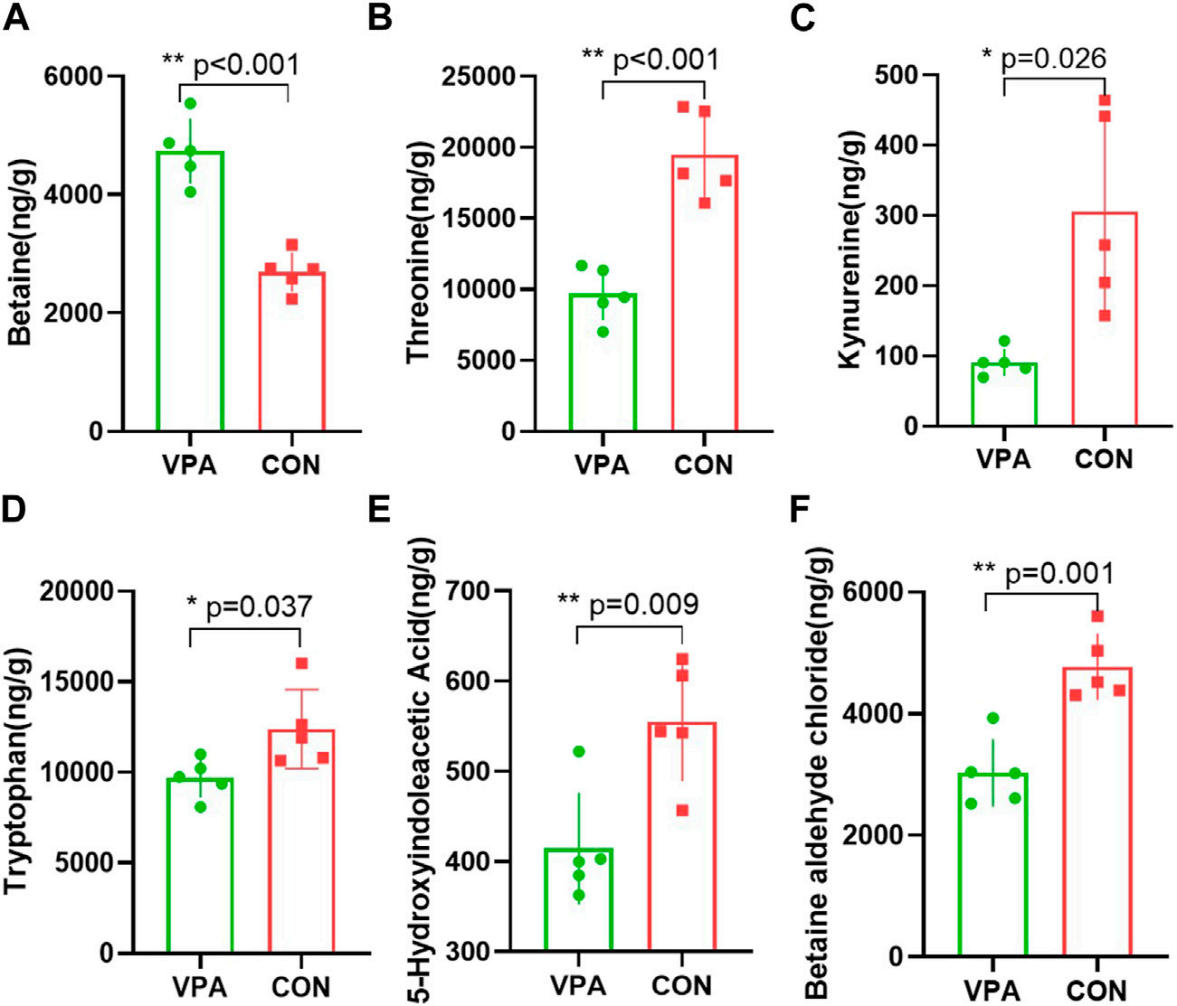
Differential neurotransmitters in the prefrontal cortex in VPA model rats. **(A)**. Betaine; **(B)** threonine; **(C)** kynurenine; **(D)** tryptophan; **(E)** 5-hydroxyindoleacetic acid; **(F)** betaine aldehyde chloride. *n* = 5/5.

Neuronal excitation/inhibition imbalance caused by neurotransmitter dysfunction is considered to be an important etiological mechanism of autism. In this study, we measured 35 neurotransmitters in the prefrontal cortex using neurotransmitter targeting technology by LC-MS. We found that five neurotransmitters (threonine, kynurenine, tryptophan, 5-HIAA, and BAC) in the prefrontal cortex of the VPA group had significantly lower levels, and betaine had a higher level than the CON group. The levels of betaine (*p* < 0.001, Figure 4A), threonine (*p* < 0.001, Figure 4B), kynurenine (*p* < 0.026, Figure 4C), tryptophan (*p* = 0.037, Figure 4D), 5-HIAA (*p* = 0.009, Figure 4E), and BAC (*p* = 0.001, Figure 4F) were significantly decreased in the VPA group compared to the levels in the CON group.

### Correlation between the differential SCFAs, neurotransmitters, and bacterial taxa

**FIGURE 5:**
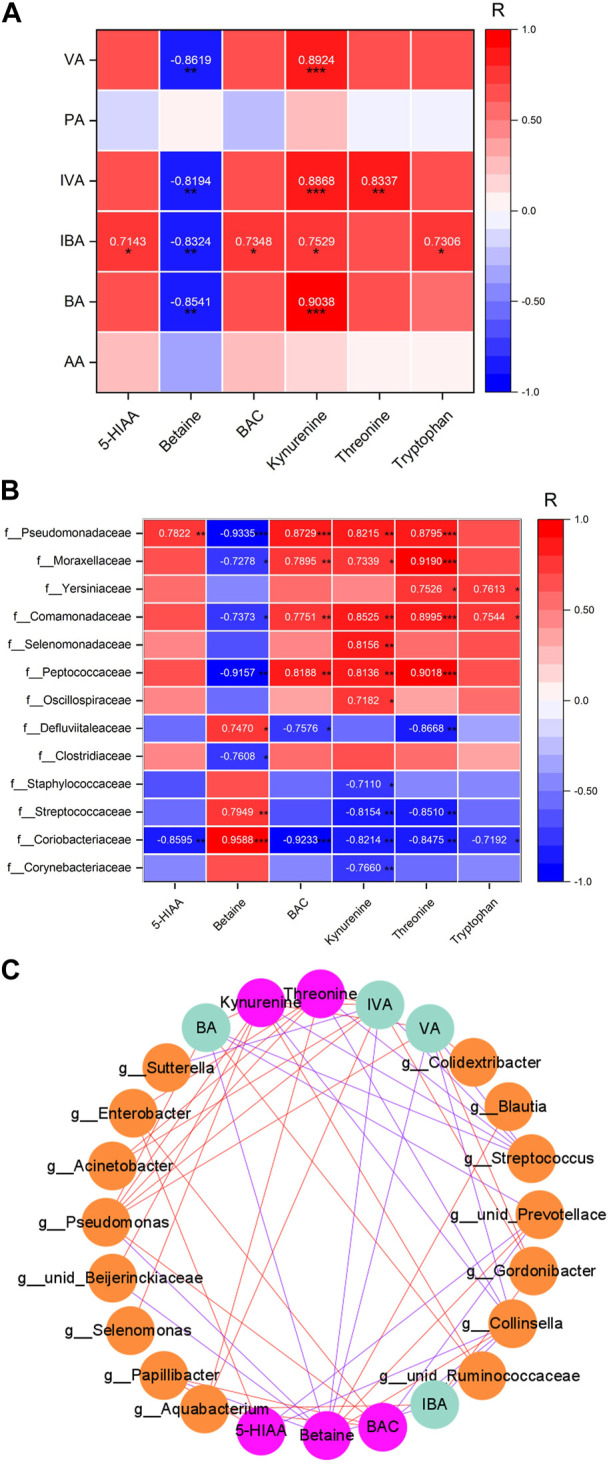
Relationship heatmap of differential SCFAs and differential neurotransmitters in the PFC. **(A)** Relationship of SCFAs and transmitters. **(B)**. Relationship of bacterial taxa and transmitters. **(C)**. Network of bacterial taxa in the genus level and SCFAs and transmitters. Figures show the correlation value |*R*| ≥ 0.7 and *p* < 0.05. *p < 0.05, **p < 0.01, and ***p < 0.001. The red line was positively correlated, and the blue line was negatively correlated. *n* = 5/5.

The relationships between the neurotransmitters and SCFAs are shown in Figure 5A. 5-HIAA was significantly positively correlated with isobutyric acid (*p* = 0.020). Betaine was significantly negatively correlated with valeric acid (*p* = 0.001), isovaleric acid (*p* = 0.004), isobutyric acid (*p* = 0.003), and butyric acid (*p* = 0.002). BAC was significantly positively correlated with isobutyric acid (*p* = 0.015). Kynurenine was significantly positively correlated with valeric acid (*p* < 0.001), isovaleric acid (*p* < 0.001), isobutyric acid (*p* = 0.012), and butyric acid (*p* < 0.001). Threonine was significantly positively correlated with isovaleric acid (*p* = 0.003). Tryptophan was significantly positively correlated with isobutyric acid (*p* = 0.016).

The relationships between the neurotransmitters and bacterial taxa are shown in Figure 5B. 5-HIAA was negatively correlated with the abundance of *Collinsella* spp. and *unidentified_Prevotellaceae* spp. (*r* = −0.859 and −0.907), while it was positively correlated with the abundance of *Papillibacter* spp. and *Enterobacter* spp. (*r* = 0.875 and 0.824). Betaine was negatively correlated with the abundance of *Papillibacter* spp., *unidentified_Beijerinckiaceae* spp., and *Pseudomonas* spp. (*r* = −0.844, −0.855, and −0.933), while it was positively correlated with the abundance of *Collinsella* spp., *unidentified_Prevotellaceae* spp., and *Blautia* spp. (*r* = 0.959, 0.913, and 0.902). BAC was negatively correlated with the abundance of *Collinsella* spp. and *unidentified_Prevotellaceae* spp. (*r* = −0.923 and −0.890), while it was positively correlated with the abundance of *Papillibacterg* spp., *Aquabacterium* spp., *Enterobacter* spp., and *Pseudomonas* spp. (*r* = 0.924, 0.865, 0.898, and 0.873). Kynurenine was negatively correlated with the abundance of *Collinsella* spp. and *Streptococcus* spp. (*r* = −0.821 and −0.810), while it was positively correlated with the abundance of *unidentified_Ruminococcaceae* spp., *Selenomonas* spp., *unidentified_Beijerinckiaceae* spp., and *Pseudomonas* spp. (*r* = 0.853, 0.816, 0.802, and 0.822). Threonine was negatively correlated with the abundance of *Collinsella* spp. and *Streptococcus* spp. (*r* = −0.848 and −0.838), while it was negatively correlated with the abundance of *Aquabacterium* spp., *Enterobacter* spp., *Acinetobacter* spp., and *Pseudomonas* spp. (*r* = 0.940, 0.827, 0.919, and 0.880).

As shown in Figure 5C, Cytoscape 3.9.1 software is used to analyze the network of relationships among the neurotransmitters, SCFAs, and bacterial taxa. Each substance is a node, forming a total of 24 nodes (15 nodes for bacterial taxa, 4 nodes for SCFAs, and 5 nodes for neurotransmitters; the correlation between nodes for |r| ≥ 0.7) and 54 edges. The more the edges connected to a node (red edge is positive regulation, and blue edge is negative regulation), the more the node is regulated by substances or regulated factors, which may be an important key node. The results showed that *Pseudomonas* spp.*, Collisella* spp., and *Streptococcus* spp. had the highest number of connecting edges among the nodes of microbial groups, which were 7, 7, and 5, indicating that these three bacterial taxa have the most extensive regulatory effects on their metabolites and/or neurotransmitters in the prefrontal lobe. Among the nodes of SCFAs, the number of connecting edges of isovaleric acid, valeric acid, and butyric acid is the largest, which is 9, 7, and 6, respectively. Among the neurotransmitter nodes, betaine, kynurenine, and threonine had the largest number of connecting edges, which were 10, 9, and 7, respectively, indicating that these three neurotransmitters are most affected by the regulation of microbiota or their metabolites.

## Discussion

A previous study revealed a tight relationship among gut microbiota, fecal metabolites, and autistic behavior in VPA-exposed offspring (Gu et al., 2022). In this study, we found that the gut microbiota, SCFAs in fecal samples, and neurotransmitters in the prefrontal cortex were significantly changed in VPA model rats compared to CON rats. A total of 13 bacterial taxa and 15 bacterial taxa were significantly decreased and increased, respectively, in VPA model rats. Five SCFAs (acetic acid, butyric acid, valeric acid, isobutyric acid, and isovaleric acid) and five kinds of neurotransmitters (threonine, kynurenine, tryptophan, 5-HIAA, and BAC) were found to be significantly decreased, whereas betaine was significantly increased in VPA model rats compared to that in the CON rats. We also found that the differential SCFAs and neurotransmitters were significantly correlated with bacterial taxa, which indicated that gut microbiota might play an important role in the pathogenesis of the VPA model of autism by regulating the level of SCFAs in fecal samples and neurotransmitters in the prefrontal cortex.

The results of this study showed that the abundance of *Bifidobacterium* spp., *Lactobacillus* spp., and *Limosilactobacillus* spp. in the VPA model group was significantly lower than that in the CON group, while the abundance of *unidentified_Prevotellaceae* spp. and *Ligilactobacillus* spp. was significantly increased. *Bifidobacterium* spp. and *Lactobacillus* spp. are considered “probiotics,” and their long-term colonization is very important for human intestinal health (Xiao et al., 2021a). *Limosilactobacillus* spp. has not only been proven to inhibit the development of colon cancer by REDOX balance (Bell et al., 2022) but also has inhibitory effects on “harmful bacteria” such as *Helicobacter pylori* and *Clostridium difficile*, so it is considered a potential “probiotic” (Engevik et al., 2020). Studies have shown that oral propionate and clindamycin can improve the concentration of Mg^2+^ in brain tissue, inhibit the growth of *Candida*, and reduce the excitotoxicity of glutamate in autism model hamsters treated with probiotics (the main components are *Bifidobacterium* and *Lactobacillus*) (El-Ansary et al., 2018). In addition, studies by Sgritta et al. (2019) showed that *Limosilactobacillus* spp. can stimulate oxytocin synthesis in a vagal-dependent manner and reverse social deficits in ASD model mice, which may be a potential microbial therapy to treat or intervene in ASD-related social dysfunction. Therefore, the results of this study suggest that VPA exposure during pregnancy can inhibit “beneficial bacteria” in rats. Prevotellaceae is considered to be the “cornerstone bacteria” of human gut. Its effects on human body are highly complex, and there is still great heterogeneity among different studies (Ley, 2016), such as anti-inflammatory or pro-inflammatory effects and metabolic effects (Kovatcheva-Datchary et al., 2015; Christensen et al., 2019; Iljazovic et al., 2021). However, Prevotellaceae species detected in this study is an unidentified genus, so its specific biological functions need to be confirmed by more studies.

Previous studies have confirmed that SCFAs are a key medium in the gut–brain axis (Silva et al., 2020), having an impact on a range of health parameters, including immunity, colonic epithelial cell integrity, and brain function and development (Barton et al., 2018; Tran and Mohajeri, 2021). In this study, we found that prenatal exposure to VPA significantly decreased the SCFA levels in fecal samples. Consistent with this finding, Liu et al. (2019) found that acetic acid and butyric acid were decreased in children with ASD. Adams et al. (2011) also found that the total SCFA level was decreased in the fecal samples of humans with autism. However, this outcome is contrary to that of Wang et al. (2012), who found that the total SCFA level was elevated in the fecal samples of children with ASD. The reason for this contradiction may be the interference of diet and other factors on gut microbiota in the population experiment, which cannot be completely avoided. However, such an interference can be reduced to a greater extent in animal experiments. In our study, we found that isovaleric acid (IVA) and isobutyric acid (IBA) were correlated with a variety of neurotransmitters. Compared to major SCFAs [propionic acid (PA), acetic acid (AA), and butyric acid (BA)], the IVA and IBA levels were much lower in both humans and animals. This may suggest that different lengths of fatty acids play different roles in tissues, regardless of the absolute content.

In an animal study, Lu et al. (Xiao et al., 2021b) found that transplanted microbiota from children with ASD modulates tryptophan and serotonergic synapse metabolism, which may lead to ASD-like behaviors in germ-free mice. In fact, the dysfunction of serotonin in blood has long been considered a biomarker of autism (Muller et al., 2016). Previously, gut microbiota was revealed to be an important driving force in the modulation of tryptophan metabolism (Gao et al., 2020). Tryptophan is a precursor of 5-HT, and it is converted to 5-HT under the catalysis of tryptophan hydroxylase 1 (TPH1) in the central nervous system or TPH2 in the periphery (Grifka-Walk et al., 2021). Meanwhile, tryptophan can also be converted to kynurenine under the catalysis of tryptophan indolamine 2,3-dioxygenase (TDO) (Grifka-Walk et al., 2021). These results may indicate that the decreases of tryptophan and kynurenine are the reason for the decrease of 5-HT in the central nervous system in autism (Muller et al., 2016). We found that 5-HIAA, which is the metabolic end product of 5-HT (Brown and Linnoila, 1990), was significantly decreased in the prefrontal cortex. In addition, betaine is a natural compound that widely exists in beetroot and seafood and has been shown to possess anti-inflammatory, anti-oxidant, and anti-apoptotic properties (Shedid et al., 2019). Huang et al. (2019) found that betaine supplementation could ameliorate autism-like symptoms by decreasing the homocysteine levels in autism model mice induced by prenatal VPA exposure since hyper-homocysteine was observed in the autism population (Kałużna-Czaplińska et al., 2013). Of note, in this study, we found that betaine in the prefrontal cortex was significantly increased in VPA model rats compared to that in control rats. This finding is contrary to Huang’s studies, which have suggested that the level of betaine in plasma samples of children with autism was decreased compared to that in control children (Hamlin et al., 2013). This may differ from the central and peripheral effects of many bioactive molecules, like 5-HT. In addition, this may indicate that higher free betaine did not bind to related enzymes (such as homocysteine) to produce beneficial biological effects. BAC is the precursor of betaine, and it is reverted to betaine under catalysis by betaine aldehyde dehydrogenase (BADH). BAC was significantly decreased in VPA model rats in the present study. Further investigation is warranted as there is no evidence to confirm the biological effect of BAC in animal and human samples. Furthermore, threonine is the only amino acid critically required for the pluripotency of mouse embryonic stem cells (mESCs), and it plays an important role in regulating the histone methylation, growth, and differentiation of stem cells (Shyh-Chang et al., 2013). The results of the present study indicate that the reduction of threonine may also be an important cause of neurodevelopmental disorders such as ASD.

In conclusion, we found that VPA exposure in the embryonic stage of development could result in gut microbiota dysfunction and decreased contents of SCFAs and alter the expression of neurotransmitters in the prefrontal cortex. We also found that five neurotransmitters were correlated with *Pseudomonas*, *Collisella*, and *Streptococcus*, at the genus level, and they were related to decreases of SCFAs (IVA, IBA, VA, and BA). According to this study, we can preliminarily infer that gut microbiota or their metabolic productions (such as SCFAs) may influence central neurotransmitter metabolism through related pathways of the gut–brain axis. Our findings also suggest that SCFA supplementation (such as butyric acid) could be considered a potential target for regulating the neurotransmitter network. Owing to the high complexity of gut microbiota, we are currently unable to explain their specific function.

## Data Availability

The data presented in the study are deposited in the NCBI repository, accession number “PRJNA862247”. Further inquiries can be directed to the corresponding author.
