# Supplementary material for: Associations between dysbiosis gut microbiota and changes of neurotransmitters and short-chain fatty acids in valproic acid model rats
Source: Front Physiol. 2023 Mar 22;14:1077821. doi: 10.3389/fphys.2023.1077821 (PMC10073564; doi:10.3389/fphys.2023.1077821)
Supplement: Supplementary file 1 [file DataSheet1.docx]

**Supplementary 1**


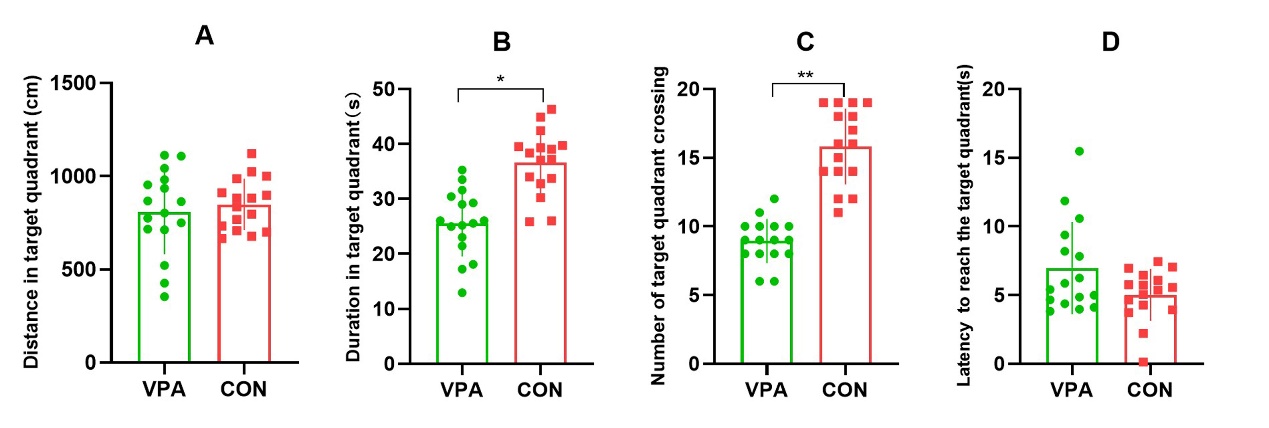


**Figure S1** **Behavioral effects of prenatal VPA exposure in Morris water maze test.** A. Distance in target quadrant. **B.** Duration in target quadrant. **C.**  Number of the target quadrant crossing. **D.** Latency to reach the target quadrant. Data expressed as mean ± SEM, *n* = 16/16. * *p* < 0.05, ** *p* < 0.001. Rats prenatally exposed to VPA (green dot and squares) compared to control (CON) group (red dots and squares). Statistical comparisons were made via *t*-test.


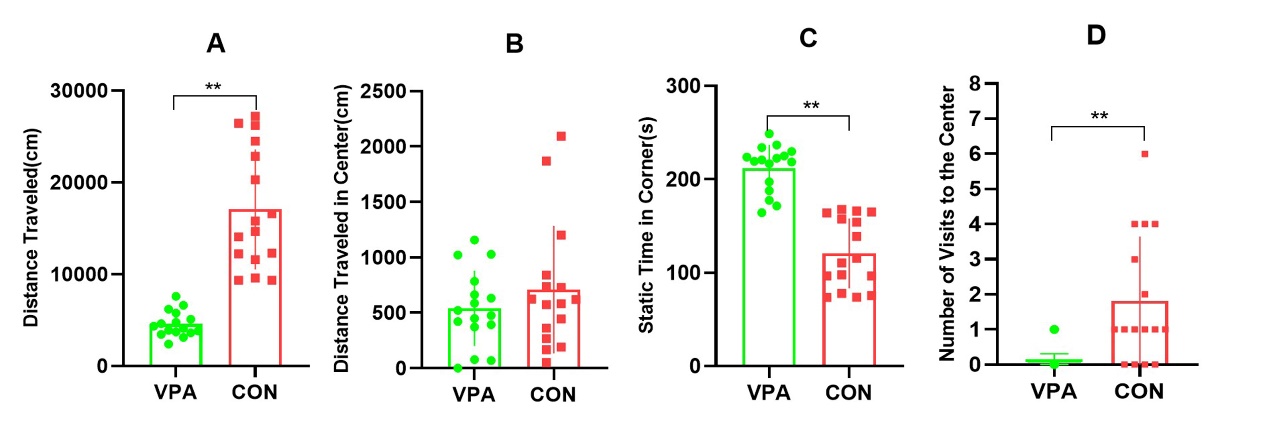


**Figure S2 Behaviors effects of prenatal VPA exposures in open field test (in 5 min).** A. Distance traveled in open field; B. Distance traveled in the center of open field; **C.** Static time in corner; D. Number of visits to the center. Data expressed as mean ± SEM, n = 16/16. * p < 0.05, ** p < 0.001 rats prenatally exposed to VPA (green dot and squares) compared to control (CON) group (red dot and squares). (*t*-test).


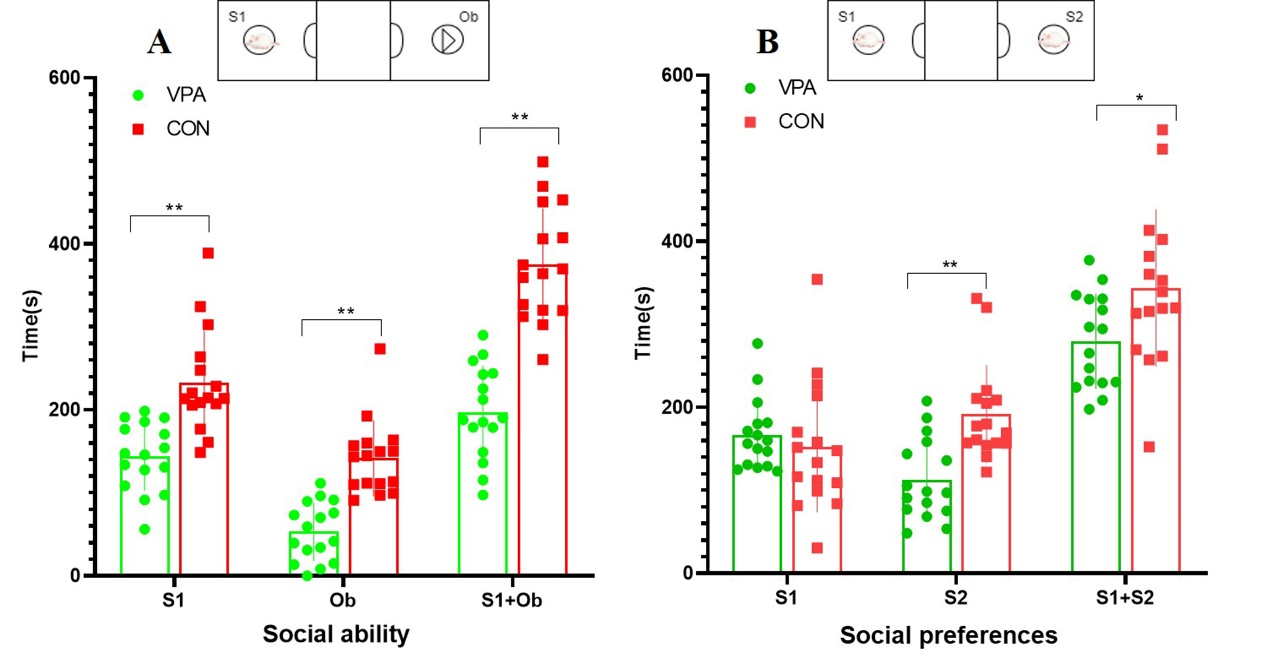


**Figure S3 Behavior effects of prenatal VPA exposures in three-chamber sociability test. A.** Social ability; **B.** Social preferences. Data expressed as mean ± SEM, n = 16/16. * p < 0.05, ** p < 0.001 rats prenatally exposed to VPA (green dot and squares) compared to control (CON) group (red dot and squares), (t-test). S1: stranger rats 1; Ob: object; S2: stranger rats 2.

**Supplementary 2**

PICRUSt2 -LEVEL2
